# Angiopoietin‐2 and hemocompatibility‐related adverse events during percutaneous left ventricular assist device supports

**DOI:** 10.1002/hsr2.211

**Published:** 2020-12-03

**Authors:** Teruhiko Imamura, Makiko Nakamura, Shigeki Yokoyama, Toshio Doi, Kazuaki Fukahara, Koichiro Kinugawa

**Affiliations:** ^1^ Second Department of Internal Medicine University of Toyama Toyama Japan; ^2^ Department of Cardiovascular Surgery University of Toyama Toyama Japan

## 1 INTRODUCTION

Despite considerable improvement in survival in heart failure patients receiving left ventricular assist devices (LVADs), hemocompatibility‐related adverse events (HRAEs) including bleeding and thromboembolic evens remain as unsolved issues.^1^, ^2^

Inappropriate activation of the inflammatory and angiogenesis cascade including angiopoietin‐2 (Ang‐2) seems to have a considerable association with the development of arteriovenous malformation and gastrointestinal bleeding during LVAD supports.^3^ Ang‐2 is considerably associated with the plasma B‐type natriuretic peptide (BNP) levels in the heart failure cohort. Furthermore, Ang‐2 is inappropriately elevated relative to BNP levels in some LVAD patients.^4^ However, its prognostic impact on HRAEs remains uninvestigated. In this study, we investigated the prognostic impact of Ang‐2 level relative to BNP (Ang‐2/BNP) obtained immediately after percutaneous LVAD implantation on future occurrences of HRAE.

## 2 METHODS

### 2.1 Patient selection

In this prospective study, consecutive patients who received percutaneous LVAD between August 2018 and February 2019 were included. Indication of percutaneous LVAD therapy is determined by the attending cardiologists. In brief, candidates had cardiogenic shock refractory to guideline‐directed medical therapy. All participants gave informed consents, and the institutional ethical review board approved this study beforehand. We affirm that this manuscript is an honest, accurate, and transparent account of the study being reported, that no important aspects of the study have been omitted, and that any discrepancies from the study as planned. The original data are available when required and considered to be appropriate.

### 2.2 Variables collection

In addition to the baseline characteristics data, Ang‐2/BNP was measured from patients' plasma within 3 days following LVAD implantation, using Human Angiopoietin‐2 Quantikine ELISA Kit.

### 2.3 Outcomes

A primary endpoint was set as any occurrence of HRAEs during LVAD supports. HRAEs consist of gastrointestinal bleeding, symptomatic stroke with image findings, and device thrombosis medically or surgically managed, according to the INTERMACS definition.^2^ Death, device explantation, or 30‐day follow‐up was censored.

### 2.4 Statistical analyses

Statistical analyses were performed using SPSS Statistics 22 (SPSS Inc, Armonk, Illinois). Continuous variables were expressed as median and interquartile.

The impact of Ang‐2/BNP on HRAE was investigated as a primary concern. Receiver operating characteristics analysis was performed to calculate a cutoff of Ang‐2/BNP for the occurrence of HRAE. Kaplan–Meier analyses and log‐rank tests were performed to compare freedom from HRAE between high Ang‐2/BNP and low Ang‐2/BNP groups. Cox proportional hazard ratio regression analyses were performed to investigate the impact of high Ang‐2/BNP on the occurrence of HRAE by adjusting for age, which is another well‐known risk factor of HRAE.

## 3 RESULTS

### 3.1 Baseline characteristics

In total, 21 patients (median 71 years old, 14 males) were included (Table 1). Ang‐2 (median 5.0 pg/mL) and BNP (median 437 pg/mL) were measured within 3 days following LVAD implantation. Median Ang‐2/BNP was 1.7 (0.9, 2.5).

**TABLE 1:** Baseline characteristics

	N = 21
Age, years	71 (59, 83)
Male sex	14 (67%)
Etiology	
Acute coronary syndrome	12 (57%)
Takotsubo syndrome	1 (5%)
Ischemic cardiomyopathy	1 (5%)
Hypertrophic cardiomyopathy	1 (5%)
Dilated cardiomyopathy	6 (28%)
Serum Ang‐2, pg/mL	5.0 (2.7, 6.3)
Plasma BNP, pg/mL	437 (264, 732)
Ang‐2/BNP	1.7 (0.9, 2.5)

*Note*: Variables are expressed as median and interquartile or number and percentage.

Abbreviations: Ang‐2, angiopoietin‐2; BNP, B‐type natriuretic peptide.

### 3.2 Ang‐2/BNP and clinical outcomes

During 6‐day (2, 15) LVAD support on median, there were seven HRAEs: four gastrointestinal bleedings, one ischemic stroke, two hemorrhagic strokes, and no device thrombosis. A cutoff of Ang‐2/BNP to predict future HRAE was calculated as 2.7 with sensitivity of 0.57 and specificity of 1.00.

**FIGURE 1:**
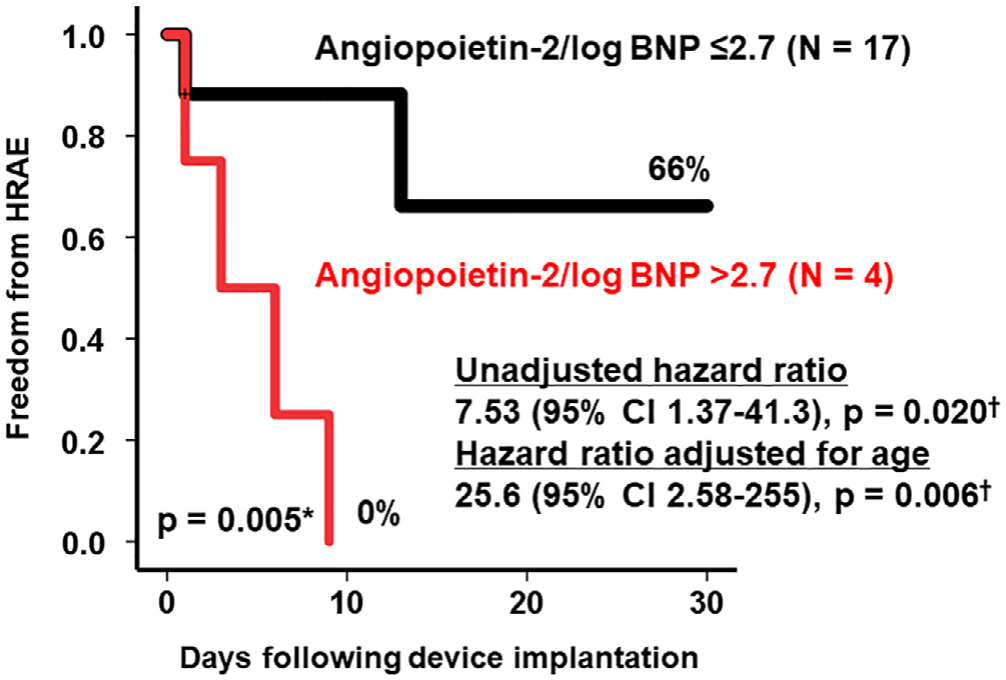
Freedom from HRAE. *P < .05 by log‐rank test; ^†^ *P* < .05 by Cox proportional hazard ratio regression analyses

Four patients had Ang‐2/BNP > 2.7: two patients had cerebral bleedings and the other two had gastrointestinal bleedings. Freedom from HRAE was significantly stratified by the Ang‐2/BNP of 2.7 (66% vs 0%, *P* = .005; Figure 1). Ang‐2/BNP was a significant risk factor of future HRAE with an unadjusted hazard ratio of 7.53 (95% confidence interval 1.37‐41.3) and an adjusted hazard ratio of 25.6 (95% confidence interval 2.58‐255).

For individual HRAEs, Ang‐2/BNP cutoff of 2.7 had sensitivity of 0.50 and specificity of 0.88 to predict gastrointestinal bleedings and sensitivity of 0.67 and specificity of 0.89 to predict strokes.

## 4 DISCUSSION

### 4.1 Ang‐2 and BNP

The inflammatory system has a considerable association with advanced heart failure: Ang‐2 and BNP have a strong correlation in the heart failure cohort.^5^ Our team recently demonstrated that Ang‐2 was inappropriately elevated relative to the BNP level during LVAD supports compared to the heart failure cohort,^4^ probably due to the stimulation of Ang‐2 activity via hematological instability.^6^ This is a rationale of why we used Ang‐2/BNP as a variable instead of Ang‐2 alone. The detailed mechanism of the variety of Ang‐2/BNP levels among each individual remains unknown.

### 4.2 Ang‐2/BNP and HRAE

The association between Ang‐2 and gastrointestinal bleeding during LVAD supports is receiving great concern. Inflammatory and angiogenesis cascade including Ang‐2 might stimulate a formation of arteriovenous malformation and increase the risk of gastrointestinal bleeding.^3^ In this study, we demonstrated that the inappropriately elevated Ang‐2 level predicted future bleedings. In other words, Ang‐2 is inappropriately activated immediately following LVAD implantation in such high‐risk patients.

Elevated Ang‐2/BNP level was associated with also the occurrence of stroke with high specificity. The detailed mechanism requires further investigations, but our team previously hypothesized the association among right ventricular failure, chronic inflammation, and stroke during LVAD supports.^7^ As a major marker of inflammation, Ang‐2 might have a critical role in the occurrence of stroke.

### 4.3 Limitations and future concerns

We should state that the study is a proof of concept, and the finding should be validated in larger scale studies. We adjusted for age alone, given its considerable impact on HRAE and small sample size,^2^ and we cannot deny any other confounders. We showed an association between Ang‐2/BNP and HRAEs, but the causality remains unknown with a lack of detailed data explaining it. We observed just for 30 days, given that the devices were percutaneous ones, and the applicability of our findings to other durable LVADs remains uncertain.

Nevertheless, our findings would give us a clue to risk‐stratify patients for the future occurrence of HRAE during LVAD supports. Furthermore, any therapeutic intervention to improve the elevated Ang‐2, including omega‐3 fatty acid or any other more specific agents,^8^ might reduce the risk of HRAE.

## 5 CONCLUSION

Elevated Ang‐2 level soon after percutaneous LVAD implantation was associated with future HRAEs. The clinical implication to intervene in Ang‐2 would be a future concern.

## FUNDING

Teruhiko Imamura receives grant support from JSPS KAKENHI: JP20K17143, which had no involvements in conducting the study and preparing the draft.

## AUTHOR CONTRIBUTIONS

Conceptualization: Teruhiko Imamura

Formal analysis: Teruhiko Imamura

Funding acquisition: Koihciro Kinugawa

Writing – original draft: Teruhiko Imamura, Makiko Nakamura

Writing – review and editing: Shigeki Yokoyama, Toshio Doi, Kazuaki Fukahara

  All authors have read and approved the final version of the manuscript.

  Teruhiko Imamura has full access to all of the data in this study and takes complete responsibility for the integrity of the data and the accuracy of the data analysis.

## TRANSPARENCY STATEMENT

Teruhiko Imamura affirms that this manuscript is an honest, accurate, and transparent account of the study being reported, that no important aspects of the study have been omitted, and that any discrepancies from the study as planned have been explained.

## Data Availability

The data that support the findings of this study are available from the corresponding author upon reasonable request.

## References

[1] Mehra MR . The burden of haemocompatibility with left ventricular assist systems: a complex weave. Eur Heart J. 2019;40(8):673‐677. 28329374 10.1093/eurheartj/ehx036

[2] Uriel N , Colombo PC , Cleveland JC , et al. Hemocompatibility‐related outcomes in the MOMENTUM 3 trial at 6 months: a randomized controlled study of a fully magnetically levitated pump in advanced heart failure. Circulation. 2017;135(21):2003‐2012. 28385948 10.1161/CIRCULATIONAHA.117.028303

[3] Tabit CE , Chen P , Kim GH , et al. Elevated angiopoietin‐2 level in patients with continuous‐flow left ventricular assist devices leads to altered angiogenesis and is associated with higher nonsurgical bleeding. Circulation. 2016;134(2):141‐152. 27354285 10.1161/CIRCULATIONAHA.115.019692 PMC4942355

[4] Nakamura M , Imamura T , Hori M , et al. Regulation of angiopoietin‐2 before and after mechanical circulatory support therapy. ASAIO J. 2020; (in press). 10.1097/MAT.0000000000001189. 32740126

[5] Link A , Poss J , Rbah R , et al. Circulating angiopoietins and cardiovascular mortality in cardiogenic shock. Eur Heart J. 2013;34(22):1651‐1662. 23349297 10.1093/eurheartj/ehs487

[6] Tabit CE , Coplan MJ , Chen P , Jeevanandam V , Uriel N , Liao JK . Tumor necrosis factor‐alpha levels and non‐surgical bleeding in continuous‐flow left ventricular assist devices. J Heart Lung Transplant. 2018;37(1):107‐115. 28651907 10.1016/j.healun.2017.06.001 PMC5722712

[7] Imamura T , Nguyen A , Kim G , et al. Optimal haemodynamics during left ventricular assist device support are associated with reduced haemocompatibility‐related adverse events. Eur J Heart Fail. 2019;21(5):655‐662. 30592363 10.1002/ejhf.1372 PMC7147872

[8] Imamura T , Nguyen A , Rodgers D , et al. Omega‐3 therapy is associated with reduced gastrointestinal bleeding in patients with continuous‐flow left ventricular assist device. Circ Heart Fail. 2018;11(10):e005082. 30354397 10.1161/CIRCHEARTFAILURE.118.005082 PMC6252056

